# Influenza: Ætiology, Symptoms and Treatment

**Published:** 1892-02

**Authors:** B. J. Nowierski

**Affiliations:** Cestohowa, Texas


For Daniel's Texas Medical Journal.

### INFLUENZA: ITS ÆTIOLOGY, SYMPTOMS AND TREATMENT.

BY B. J. NOWIERSKI, M. D., CESTOHOWA, TEXAS.

Read at meeting West Texas Medical Association, and ordered sent to the JOURNAL—official organ.

THIS DISEASE is known to every country of the globe, as all places have had one or more epidemics of this disorder. It is known by many names. Influenza was first used in Italy in the seventeenth century, from some supposed influences this disease had from the stars. About the year 1743, the disease was named la grippe, derived from the Polish word *grypka*, by the French. Both these names are now used in medical literature:

The ætiology of the disease at the present time is unknown. Some few writers believe that the cause is a specific microbe, yet most writers do not agree to this statement, and claim that they find two or more different microbes; yet no one specific microbe can be demonstrated the cause of influenza. Bouchard writes: "He does not believe that there is any specific microbe the cause of this disease, as he has found three different species in it.

His opinion is that some one of the ordinary microbes—different in different cases,—acting upon a person whose organism has lost the power of resistance, becomes pathogenic and produces the disorder." (A. J. of M. S., Vol. 99.) Others claim that the trouble is due to some malarial origin, or from fungi; yet it has only been shown that in low, malarial districts, influenza sometimes assumes grave or malignant character, but nothing to prove that malaria or fungi produced the disease. No one has proved that either soil or atmospheric changes causes this disorder, as all places and in all changes epidemics of influenza have occurred. If any special atmospheric condition is the cause of influenza, I can only believe with Dr. R. Parsons, who says: "Between the epidemics and the condition of the atmosphere, there appears to be a connection, different from that which depends on a mere alteration of temperature, or of dryness or moisture." What this connection or difference is, he does not state, thus leaving a doubt if influenza can be caused by such a condition. The cause of this disease, to the present time, is in dispute, and, whether due to all or any one of the above theories, is a point that is to be learned by future investigation.

The symptoms of influenza depend on the type of the disorder; one case will have a high continued temperature, another a more severe type of the specific catarrh of the nose and throat, others complain principally of the rheumatic pains, while some cases become complicated; so that no one and the same symptom will always be found alike in all cases. Influenza generally manifests itself by a high temperature and more or less of a specific catarrhal condition of the nose, mouth, throat, and bronchitis; it is generally ushered in by a sneezing, a coldness down the spine or a chill, with flashes of heat, a dry skin, quick pulse and a severe headache. As the disease continues, the temperature ranges from 100 to 104 or 5 degrees—the duration from three to seven days—pulse 100 or more, with severe frontal headache, giddiness, swimming of the head, pains in back, calves and thighs, a general muscular weakness, mental depression, appetite impaired; some have vomiting and diarrhœa, others are constipated. Some cases have a dry cough, but bronchitis, with a thick tenacious mucous expectoration, occurs in the majority of cases. Albumen in the urine, with hyaline and epithelial casts; also, retention of urine in elderly persons are of occurrence.

These are the principal symptoms, though others occur when influenza becomes complicated with other diseases, as pneumonitis, pleuritis, pericarditis, etc., in which case the influenza is of graver character, and the prognosis with a complication is unfavorable, while otherwise, the prognosis, except in very old or young cases, is favorable, and under proper treatment, recovery takes place.

In the treatment of influenza, many medicines are used, and nearly every physician has a special line of treatment, which he claims to be the most successful, with him; and, in order to find the one most successful, I will quote some of the especial treatments.

Dr. Huchard uses quinine, in combination with ext. of aconite and cinchona, to allay the periodic and febrile element. If neuralgia is prominent he uses hydrobromate of quinine with aconite; for the bronchitis, he uses powd. ipecac and opium, powd. squills and quinine. Dr. R. Batholo recommends, at the beginning, calomel, gr. ij at night, with the inhalation of some vapor, as of creosote, turpentine, or carbolic acid, with steam, or to insufflate some powder, such as salicylate of bismuth, tannin, or resorcin. For internal remedies, he would use atrophia in solution gr. j to ℥j of water, dose from 1 to 5 drops; for the pain, antipyrin or acetanilid; and salicylate of cinchona and quinine as a prophylactic remedy. Dr. Dobell recommends the inhalation of creosote, oil of cloves, and eucalyptus and camp. tinct. opium with boiling water. Internally, he uses spir. of camphor, nitrous spirits of ether and comp. tinct. cinchona. If the temperature rises over 101 degrees, then he uses antifebrin and applies hot poultices if oppression of the chest sets in. Dr. Allison's principal remedy seems to be tannic acid in doses of gr. xx to xxv daily. Dr. Blackwood used electricity, claiming that these means, with little or no medication, he has had excellent results and cured his cases with from two to six applications. Dr. DaCosta treats his cases according to the symptoms, dry cupping some with benefit and using such medicines as to him each individual case needs; he also states that, although the journals of the present time are full of different treatments, he is unable to find one that is suitable to every case of this disease.

These are a few of the special treatments that I found in the journals at my command, and which one, if any of them, is the best, I will leave for others to decide.

The treatment I found most successful in uncomplicated cases, is the using of quinine and antikamnia, acetanilid or antipyrin (if patient is of robust constitution), in connection with aconite to control the temperature. As a gargle, I use a saturated solution of chlorate of potash or boracic acid and glycerine; as expectorant, using ipecac, squills, and senega and chloride of ammonia. As soon as the fever declines I use tonic of iron, quinine and strychnia or arsenic.

This treatment, I do not claim to cure all cases of influenza; it is merely one that I use, and with which I have had success.

